# Different substrate specificities of the two ADPR binding sites in TRPM2 channels of *Nematostella vectensis* and the role of IDPR

**DOI:** 10.1038/s41598-019-41531-4

**Published:** 2019-03-21

**Authors:** Frank J. P. Kühn, Joanna M. Watt, Barry V. L. Potter, Andreas Lückhoff

**Affiliations:** 1 Institute of Physiology, Medical Faculty, RWTH Aachen, D52057 Aachen, Germany; 2 Medicinal Chemistry and Drug Discovery, Department of Pharmacology, University of Oxford, Mansfield Road, Oxford, OX1 3QT UK; 3 Wolfson Laboratory of Medicinal Chemistry, Department of Pharmacy and Pharmacology, University of Bath, Claverton Down, Bath, BA2 7AY UK

## Abstract

NvTRPM2 (*Nematostella vectensis* Transient Receptor Potential Melastatin 2), the species variant of the human apoptosis-related cation channel hTRPM2, is gated by ADP-ribose (ADPR) independently of the *C*-terminal NUDT9H domain that mediates ADPR-directed gating in hTRPM2. The decisive binding site in NvTRPM2 is likely to be identical with the *N*-terminal ADPR binding pocket in zebra fish DrTRPM2. Our aim was a characterization of this binding site in NvTRPM2 with respect to its substrate specificity, in comparison to the classical ADPR interaction site within NUDT9H that is highly homologous in hTRPM2 and NvTRPM2, although only in NvTRPM2, catalytic (ADPRase) activity is conserved. With various ADPR analogues, key differences of the two sites were identified. Particularly, two reported antagonists on hTRPM2 were agonists on NvTRPM2. Moreover, IDP-ribose (IDPR) induced currents both in hTRPM2 and NvTRPM2 but not in NvTRPM2 mutants in which NUDT9H was absent. Thus, IDPR acts on NUDT9H rather than *N*-terminally, revealing a regulatory function of NUDT9H in NvTRPM2 opposed to that in hTRPM2. We propose that IDPR competitively inhibits the ADPRase function of NUDT9H and evokes ADPR accumulation. The findings provide important insights into the structure-function relationship of NvTRPM2 and will allow further characterization of the novel ADPR interaction site.

## Introduction

In metazoans there are currently five ion channels known that have a built-in enzyme domain and are therefore named “chanzymes”. Four of these belong to the Melastatin subfamily of Transient Receptor Potential cation channels^1–5^. The functional role of the respective enzyme domain is quite different in the individual chanzymes.

In the human TRPM2 channel (hTRPM2), the presence of the NUDT9H (Nudix hydrolase 9 homology) domain is essential for ADPR-dependent channel activation while the ADPRase function is negligible^2,6–9^. In contrast, in the species variant NvTRPM2 from the sea anemone *Nematostella vectensis*, the ADPRase function is intact and the NUDT9H domain is not necessary for ADPR-mediated channel gating^10^. These findings directly imply the presence of a novel interaction mode between ADPR and channel protein^10,11^ which must be different from NUDT9H-related ADPR binding^12,13^ and furthermore from mechanisms like ADP-ribosylation^14^.

Very recently, the essential role of ADPR binding outside of the NUDT9H domain has been stressed in TRPM2 of the zebra fish *Danio rerio* (DrTRPM2)^15^. In this cryogenic electron microscopy (cryo-EM) study, an *N*-terminally localized ADPR binding pocket was demonstrated crucial for gating. The critical amino acid residues are largely conserved in hTRPM2 as well as in NvTRPM2. On the other hand, no findings were obtained in DrTRPM2 that would indicate ADPR binding to NUDT9H or conformational changes induced by such binding. This should not come to a surprise because the DrTRPM2 channel studied almost exactly corresponds to the delta-C splice variant of hTRPM2^16^, i.e. it is lacking a stretch of 32 amino acid residues the removal of which abolishes the ADPR-sensitivity of hTRPM2 completely^7^. Even more recently, a further cryo EM study was reported on hTRPM2^17^. In contrast to the situation in DrTRPM2, here it was demonstrated that conformational shifts consistent with gating are induced by ADPR binding to the NUDT9H domain. Thus, many previous studies were confirmed that proposed the essential role of this domain in hTRPM2 because already subtle mutations in this region lead to dysfunctional channels, e.g. refs^7,8,12^. A partial cryo EM structure of the NvTRPM2 channel is also available, unfortunately without information on the NUDT9H domain (Zhang *et al*.)^18^. We have provided functional evidence^11^ that the enzymatic function of NUDT9H may play a role as regulator of the ADPR availability in NvTRPM2. In view of the divergent data on both ADPR binding sites in various TRPM2 orthologues, information about their substrate specificities may be of immense help to disentangle their species-specific functions.

The human TRPM2 orthologue is involved in oxidative-stress mediated apoptosis and induces a massive long-lasting influx of Ca^2+^ into the cell^16,19,20^. Since intracellular Ca^2+^ potentiates ADPR-induced gating, there is a positive feedback mechanism which enables the typical whole-cell current kinetics with slow onset, gradually raising amplitude and little inactivation. These characteristics become especially apparent under more physiological conditions^21–23^ of intact human cells with moderate intracellular concentrations of ADPR and Ca^2+^ (e.g. refs^2,5,18,24–26^) For NvTRPM2, an essential role of Ca^2+^ has not only been demonstrated for ADPR-directed gating^5,18^ but also for channel activation by the Ca^2+^ channel modulator 2-Aminoethoxydiphenyl borate (2-APB)^25^. However, the kinetics of ADPR-dependent currents of NvTRPM2 are distinctly different from hTRPM2. The channel opens abruptly and achieves maximum currents within seconds and then rapidly undergoes an almost complete inactivation. The channel seems to work on the “all-or-nothing principle”. Once a threshold of intracellular ADPR concentration is reached, peak currents develop with amplitudes that are hardly related to the ADPR concentration, provided Ca^2^+ is present at least at one side of the cell membrane^5^. Hence, the dose-response relationship for ADPR does not show further increases of the responses once the threshold^5^ is surpassed, which is similar to the situation in hTRPM2, with the exception that far lower ADPR concentrations are required in NvTRPM2. The different kinetics and required agonist concentrations in both orthologous channels may give rise to speculation on the biological function of NvTRPM2, since they seem to serve better for short-lasting signal transduction cascades than the induction of terminal reactions like oxidative-stress-induced apoptosis in mammals^11^.

The agonist specificity of human TRPM2 for various ADPR-analogues has been intensively examined^13,26–31^. The spectrum of action of these analogues ranges from effective inhibitors (e.g. 8-(thiophen-3-yl)-ADPR, 8-(3-acetylphenyl)-ADPR to the super-agonist 2′-deoxy-ADPR^27,30^. For NvTRPM2 so far only ADPR-2′-phosphate was shown to be similarly effective as ADPR and facilitates channel activation independently of the NUDT9H domain^10^.

Therefore, the aim of the present study was a comprehensive analysis of the agonist-specificities of the additional ADPR binding site in NvTRPM2, especially in comparison to the conventional NUDT9H domain. These experiments should lead to a better understanding of the ADPR-driven mechanisms of channel activation; additionally, they may provide further information about the specific regulatory function of the NUDT9H domain in NvTRPM2.

For this purpose, we performed whole-cell patch clamp experiments of the two TRPM2 orthologues heterologously expressed in HEK-293 cells. Additionally, the NvTRPM2-∆NUD variant (in which the NUDT9H domain is removed but ADPR-dependent channel function preserved) was employed to disentangle potential secondary effects of ADPR analogues to those on the NUDT9H domain through interference with ADPR degradation.

## Results

### Different current kinetics of the two TRPM2 orthologues from man and sea anemone

**Figure 1:**
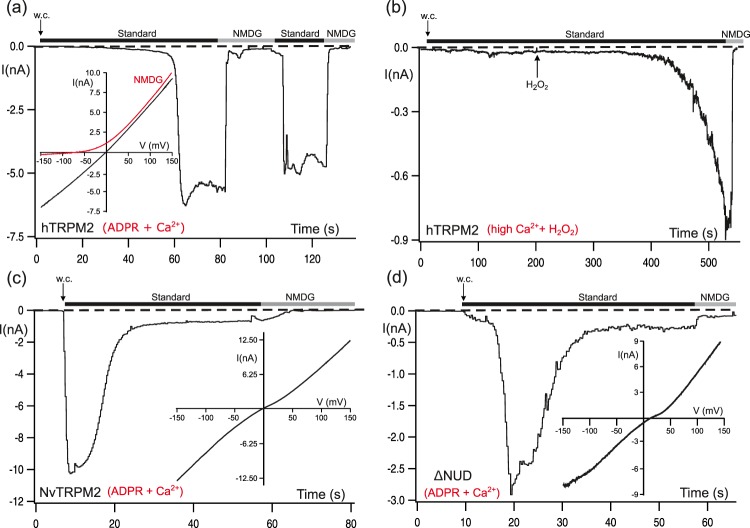
Typical kinetics of ADPR-dependent currents of hTRPM2 and two variants of NvTRPM2. Representative whole-cell patch-clamp experiments of HEK-cells expressing hTRPM2, NvTRPM2 and NvTRPM2-ΔNUD as indicated. (**a**) hTRPM2: ADPR (150 µM) was infused into the cell through the patch pipette together with 1 µM Ca^2+^. Substitution of external Na^+^ in the standard bath solution (ref. to Methods section) with the impermeable cation NMDG (indicated with bars) blocks the inward currents. Note the delayed time course of activation as well as the absence of a relevant channel inactivation during several minutes. The corresponding current-voltage relation, as obtained with voltage-ramps, is given in the inset. (**b**) hTRPM2: the pipette solution contained an excessively high concentration (1 mM) of Ca^2+^ but no ADPR. Activation is only induced after extracellular application of 10 mM H_2_O_2_ (as indicated). (**c**) NvTRPM2: The pipette solution contained 150 µM ADPR and 1 µM Ca^2+^. The corresponding current-voltage relation is given in the inset. The onset of currents started immediately after reaching whole-cell configuration (w.c.). Maximum currents developed within a few seconds followed by a rapid current decay. Small remaining inward currents can be blocked with extracellular NMDG solution. (**d**) NvTRPM2-ΔNUD: The pipette solution contained 150 µM ADPR and 1 µM Ca^2+^. The corresponding current-voltage relation is given in the inset. There is a small but consistent delay of current onset. All experiments were repeated at least five times confirming the results.

The two channel orthologues, hTRPM2 and NvTRPM2, displayed distinct characteristics under stimulation with the common agonist ADPR (Fig. 1, see also refs^5,10^). When ADPR (150 µM along with a Ca^2+^-concentration of 1 µM) was intracellularly applied through the patch pipette to hTRPM2 expressing HEK-cells, a current developed gradually and reached a maximum after about one minute (Fig. 1a). The current-voltage relation was almost linear, but the inward component could be blocked by substitution of extracellular cations with the impermeable cation NMDG (inset of Fig. 1a). There was very little inactivation of the current that remained nearly constant over several minutes. In Fig. 1b different modes of channel stimulation were tested. Ca^2+^ alone, even at the excessive intracellular concentration of 1 mM, did not lead to channel activation. Extracellular hydrogen peroxide (H_2_O_2_, 10 mM) induced currents consistently, although only after a characteristic delay of several minutes. These results have been explained previously by intracellularly accumulated ADPR, which is most probably released during oxidative-stress mediated apoptosis (e.g. ref.^8^). Similar effects of H_2_O_2_ occurred at a Ca^2+^-concentration of 1 µM as well, but Ca^2+^ alone did not induce currents through hTRPM2 even if considerably more time is allowed than shown in Fig. 1b.

When ADPR was applied to NvTRPM2 (Fig. 1c) in the same way as to hTRPM2 (Fig. 1a), currents developed almost immediately, reaching maximum amplitudes within less than 5 s. Afterwards, a rapid inactivation took place that reduced the current amplitude to less than 15% within 30 s. Again NMDG blocked the inward component of the current (Fig. 1c). In standard bath solution (145 mM Na^+^, 1.2 mM Ca^2+^, 1.2 mM Mg^2+^), inward and outward currents were equal in amplitude; the small deviation from linearity of the IV curve was consistently observed but has not been characterized so far by further experiments.

Closely similar current kinetics after stimulation with ADPR were also observed in a truncated variant of NvTRPM2 where the *C*-terminal ADPRase domain (NUDT9H) had been removed^10^. In this variant, NvTRPM2-ΔNUD, current activation was slightly delayed (but was still much faster than in hTRPM2) and the peak amplitudes were reduced, if compared to the full-length channel (ref.^10^, compare also Fig. 1c,d). These features are not caused by a reduced surface expression^10^ but are possibly related to a suboptimal stability of the artificially truncated channel protein.

### Consistent agonist specificities of the two TRPM2 species variants

**Figure 2:**
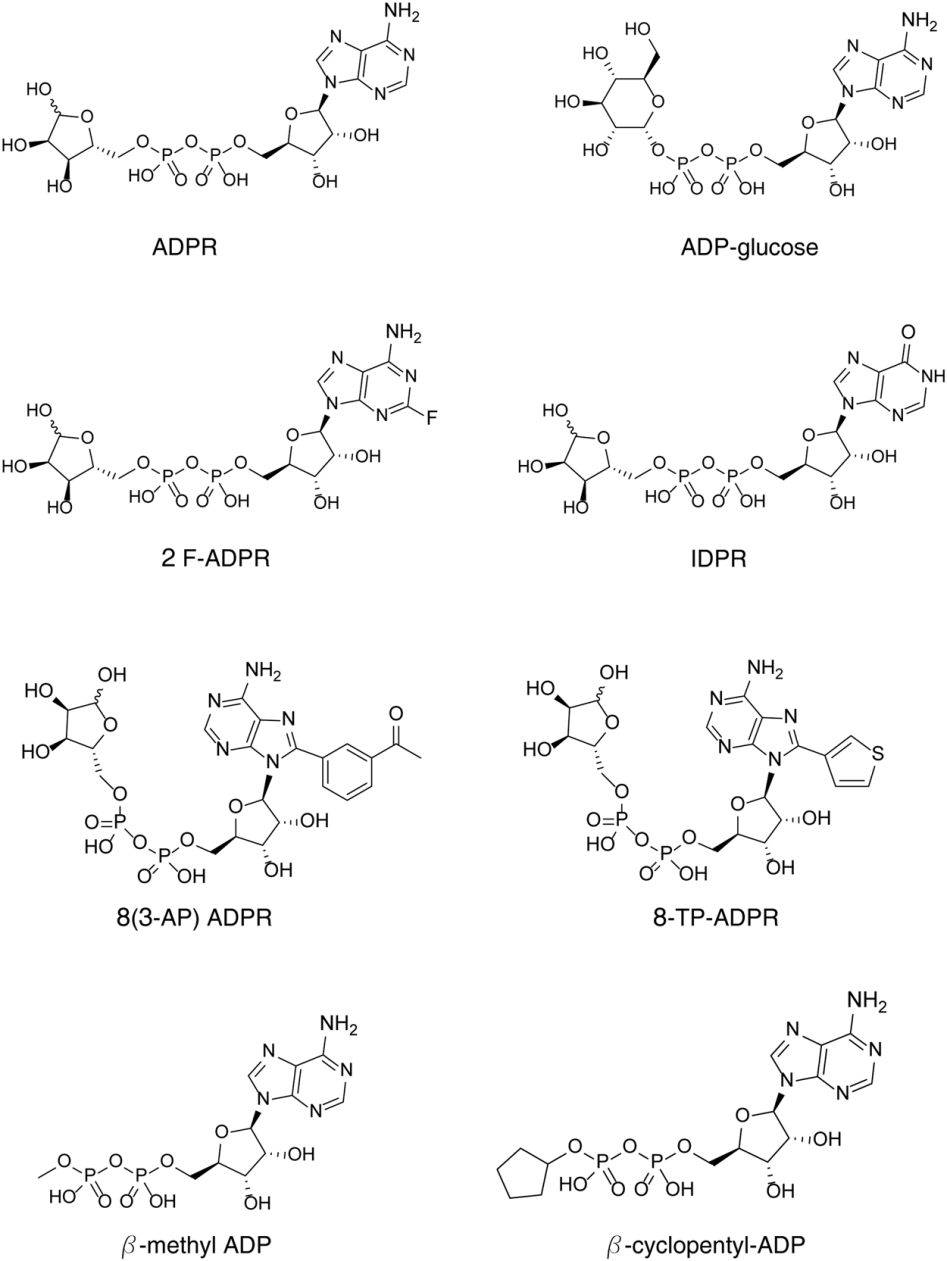
Structures of ADPR analogues. Synthetic ADPR analogues were modified in either the terminal ribose (ADP-glucose, β-methyl-ADP, β-cyclopentyl-ADP) or adenosine [2F-ADPR, IDPR, 8-(3-AP)-ADPR, 8-TP-ADPR] motif.

In a recent cryo EM study^18^, a partial structure of NvTRPM2 was revealed at high resolution but intriguing questions about the second ADPR binding site^10^ and its interaction with the other channel domains remained unanswered^18^. For a functional characterization, we investigated the activating properties of various, primarily synthetic, analogues of ADPR, with a focus on those that are modified in the terminal ribose and adenosine motifs (Fig. 2).

**Figure 3:**
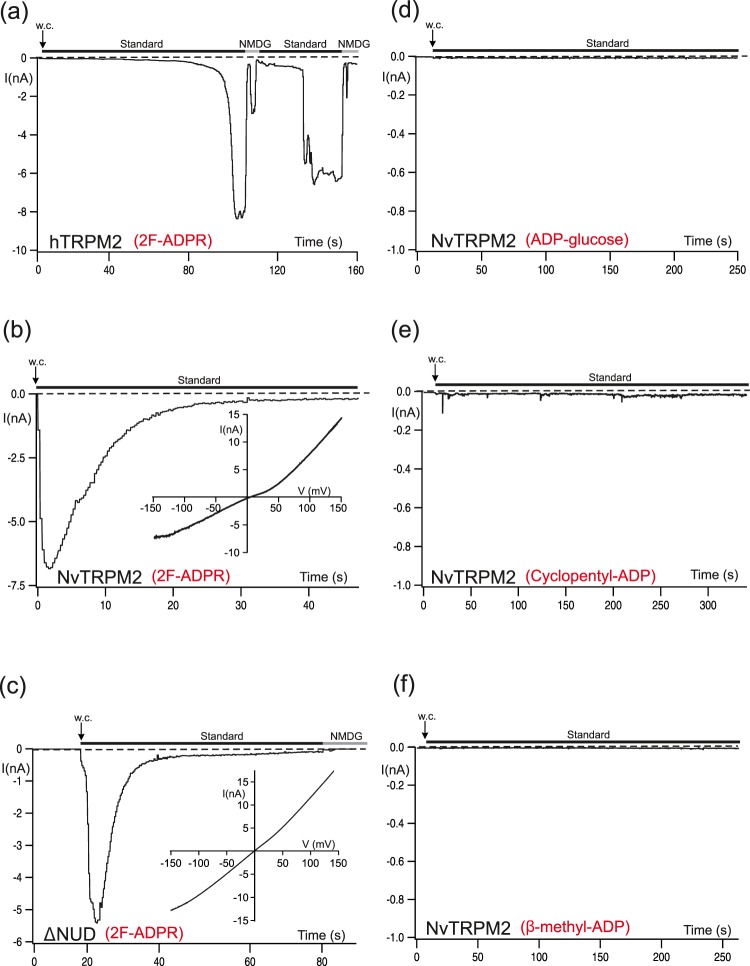
Corresponding effects of the ADPR-analogues 2-F-ADPR, ADP-glucose, β-cyclopentyl-ADP and β-methyl-ADP on hTRPM2 and NvTRPM2 variants. (**a**–**c**) Representative whole-cell patch-clamp experiments of HEK-cells expressing hTRPM2, NvTRPM2 and NvTRPM2-ΔNUD as indicated. Stimulations were performed with a pipette solution containing 2F-ADPR (300 µM) along with 1 µM Ca^2+^. Corresponding current-voltage relations are given in the insets. (**d–f**) Stimulation of HEK-cells expressing NvTRPM2 with three different ADPR-analogues (as indicated, each at 300 µM) characterized by distinct modifications of the terminal ribose unit. All experiments were repeated at least five times confirming the results.

In hTRPM2, the derivative with a fluorine atom in the adenine 2-position (2F-ADPR) acts like ADPR although it is a weaker agonist^27,30^, while ADP-glucose, β-cyclopentyl-ADP, β-methyl-ADP are without effect^13,30^. In our hands 2F-ADPR activated hTRPM2, as well as both variants of NvTRPM2, in a very similar way to ADPR (Fig. 3a–c). This is not too surprising, as 2F-ADPR is designed sterically to be very similar to ADPR, with the 2-fluoro group added only to modulate the electronics of the adenine ring^32^. Similar results have been obtained in an earlier study using the ADPR analogue ADPR-2′-phosphate^10^. Hence, there is no essential difference between 2F-ADPR, ADPR-2′-phosphate and ADPR with respect to their effects on two different ADPR binding sites. For 2F-ADPR the result indicates that the *N*1 nitrogen atom of the purine ring, adjacent to the *N*6-amino group, is probably playing no role in H-bonding to the protein, as this nitrogen atom is very strongly deactivated through the adjacent fluorine substitution, with a pK_a_ lowered by several orders of magnitude^32^. Likewise, no differential effect on either binding site was demonstrated for ADP-glucose, β-cyclopentyl-ADP and β-methyl-ADP, each of them modified diversely at the terminal ribose position. None of these ADPR derivatives stimulated currents in NvTRPM2 (Fig. 3d–f) at concentrations that are almost saturating for ADPR^10^. These results are completely in line with those^13,30^ on hTRPM2. Thus, the structure of the terminal ribose moiety is crucial for the agonistic effect of ADPR on both TRPM2 channel orthologues.

### Opposite agonist specificities of the two TRPM2 species variants

**Figure 4:**
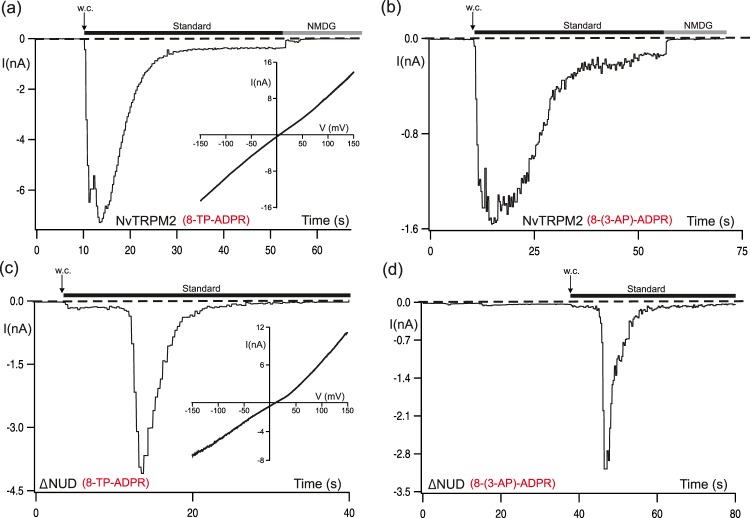
Activation of NvTRPM2 and NvTRPM2-∆NUD by 8-(thiophen-3-yl)-ADPR and 8-(3-acetylphenyl)-ADPR, established antagonists on hTRPM2. (**a**,**b**) Representative whole-cell patch-clamp experiments of HEK-cells expressing wild-type NvTRPM2. Stimulations were performed by infusion of the cells with a pipette solution containing 8-(thiophen-3yl)-ADPR (150 µM) or 8 (3-acetylphenyl)-ADPR (150 µM) together with 1 µM Ca^2+^. (**c,d**) Similar experiments as shown in panels a and b but with HEK-cells expressing NvTRPM2-∆NUD. Note the characteristic delay of current onset in NvTRPM2-∆NUD. Corresponding current-voltage relations are given in the insets. For statistics see Fig. 5.

**Figure 5:**
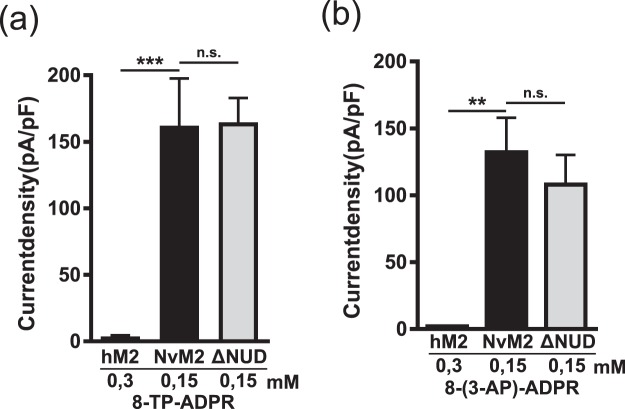
Comparison of the activation properties of 8-(thiophen-3-yl)-ADPR and 8-(3-acetylphenyl)-ADPR on hTRPM2, NvTRPM2 and NvTRPM2-∆NUD. Relations of current densities to the applied concentration (as indicated) of the ADPR-analogues 8-(thiophen-3yl)-ADPR (**a**) and 8-(3-acetylphenyl)-ADPR (**b**) obtained from whole-cell patch-clamp recordings of cells transfected with either hTRPM2, NvTRPM2 or NvTRPM2-∆NUD (as indicated). All data are presented as mean ± s.e.m. Differences are significant at **P < 0.01 ***P < 0.001, evaluated with one-way ANOVA and the Bonferroni correction, n = 3–13. n.s., not significant.

Next we tested on NvTRPM2 two ADPR analogues that have been shown to be strong antagonists of ADPR-dependent channel gating on human TRPM2: 8-(thiophen-3-yl)-ADPR (IC_50_ for hTRPM2 of 51 µM) and 8-(3-acetyl-phenyl)-ADPR (IC_50_ for hTRPM2 of 49 µM)^27^. Either substance was applied, as usual, through the patch pipette at a concentration of 150 µM, along with 1 µM Ca^2+^. Both ADPR derivatives activated the NvTRPM2 channel, both the full-length variant (Fig. 4a,b) and the truncated version without the NUDT9H-domain (Fig. 4c,d). A closer inspection of the current kinetics in NvTRPM2-∆NUD revealed a short delay of current onset (Fig. 4c,d) which occurs as well with ADPR as stimulus (see Fig. 1d). These data, therefore, demonstrate an opposite agonist specificity of both species variants of TRPM2 (summarized in Fig. 5) which is manifested when the adenosine ring of ADPR is modified at the 8-position. This is interesting as substitution with such bulky groups at the adenine 8 position often changes the *syn-anti* conformation of the adenosine base (see structures in Fig. 2) and this has been invoked in attempting to rationalize the antagonistic effects of *e.g*. 8-bromo-ADPR at hTRPM2^27^. This is not expected for 2F-ADPR, where the base conformation should be similar to ADPR itself.

### IDPR-mediated activation of hTRPM2 and NvTRPM2

Since the characterization of the novel ADPR binding site of NvTRPM2 is always concerned with its significance for the situation *in vivo*, we also extended our focus to encompass inosine 5′-diphosphate ribose (IDPR). This ADPR-analogue possesses a small modification of the adenine ring at *C*−6, effectively equivalent to an *N*6-deamination of ADPR, and has so far not been attributed with a physiological role in mammalian cells^33^, but this might be different in far distantly related organisms like *Nematostella vectensis*. Again, like 2F-ADPR, this modification is not expected to influence the adenosine base conformation from that in ADPR.

Together with ADPR, IDPR is the only substrate of the human Nudix hydrolase NUDT9^33^. It should be noted that this was shown using relatively high concentrations (300 µM) of IDPR. When tested on human TRPM2 by others, IDPR did not activate the channel at a concentration of 100 µM^30^. On the other hand, IDPR showed no antagonistic effects on hTRPM2, because at a concentration of 900 µM it failed to inhibit the stimulation of hTRPM2 by 100 µM ADPR^27^.

**Figure 6:**
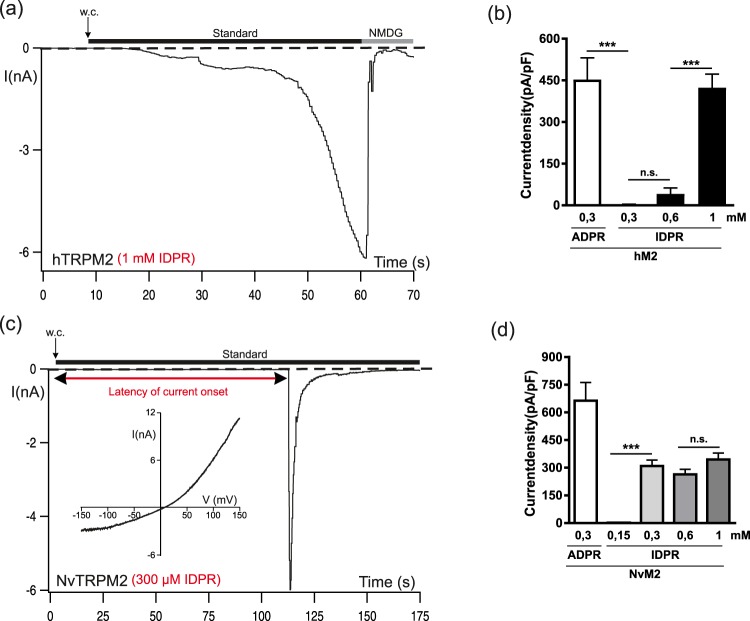
High concentrations of IDPR activate hTRPM2 and NvTRPM2. (**a**) Stimulation of HEK-cells expressing hTRPM2 with high concentrations of IDPR (1 mM) and 1 µM Ca^2+^ in the pipette solution. Note the delayed time course of current development which is indistinguishable from that under stimulation with ADPR (see Fig. 1(a,b) Summary of the effects of IDPR on hTRPM2 including control experiments with ADPR. All data are presented as mean ± s.e.m. Differences are significant at ***P < 0.001 evaluated with a one-way ANOVA and the Bonferroni correction, n = 6–14. n.s., not significant (**c**) Stimulation with IDPR (300 µM) of HEK-cells expressing NvTRPM2. Note the characteristic delay of current onset (indicated in the figure with a red double arrow) which is about 1–2 minutes, as well as the very rapid current decay. The corresponding current-voltage relation is given in the inset. (**d**) Summary of the effects of IDPR on NvTRPM2 with ADPR control included. All data were presented as mean ± s.e.m. Differences are significant at ***P < 0.001 evaluated with an unpaired Student’s t-test, n = 6–16. n.s., not significant.

**Figure 7:**
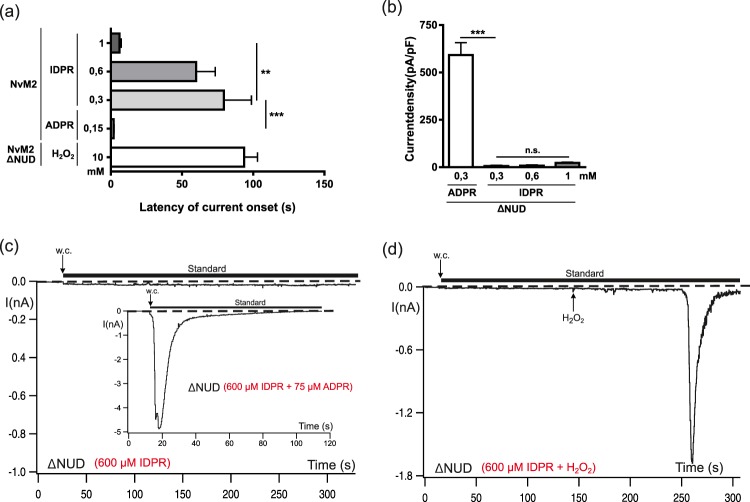
Delayed onset of IDPR-induced currents in NvTRPM2 and absence of IDPR-induced effects on NvTRPM2-∆NUD. (**a**) Comparison of the latencies of current onset after stimulation of HEK-cells expressing NvTRPM2 with ADPR, H_2_O_2_ or increasing concentrations of IDPR (as indicated). (**b**) Summary of the effects of IDPR on NvTRPM2-∆NUD with ADPR control included. For panels a and b all data are presented as mean ± s.e.m. and differences are significant at **P < 0.01 and ***P < 0.001 evaluated with a one-way ANOVA and the Bonferroni correction, n = 5–15. n.s., not significant (**c**) No currents were elicited with IDPR (600 µM) in HEK-cells expressing NvTRPM2-∆NUD. In a control experiment (inset), 75 mM ADPR was additionally added to the pipette solution, resulting in typical ADPR-induced currents. (**d**) Characteristic currents through NvTRPM2-∆NUD were also evoked by extracellular stimulation with 10 mM H_2_O_2_ (indicated by an arrow) in spite of the presence of 600 µM IDPR in the pipette solution.

Since so far no comprehensive analysis has been performed to examine the potential agonistic properties for IDPR on either hTRPM2 and NvTRPM2, we decided to use higher concentrations of IDPR (300 µM to 1 mM in the presence of 1 µM Ca^2+^) in order to test the sensitivity of both channel orthologues (Figs 6 and 7). At 300 µM, IDPR was insufficient on hTRPM2 (n = 11) for activation, as it was in most experiments at 600 µM. But as it displayed typical channel activation in n = 2 out of 14 experiments, we increased its concentration to 1 mM, when it then consistently evoked large currents on hTRPM2 (Fig. 6a,b; n = 6) that were indistinguishable from ADPR-induced currents with respect to amplitude and current kinetics (see Figs 1a and 6a). As a control, no currents were elicited in the hTRPM2-ΔNUD variant with 1 mM IDPR in the pipette solution (n = 8). Moreover, we did not observe inhibitory effects of IDPR on ADPR-induced currents of full-length hTRPM2, neither when ADPR (75 µM) and IDPR (600 µM) were infused together (n = 2), nor when the pipette solution contained only IDPR (300 µM) and stimulation was performed with H_2_O_2_ (n = 3).

In NvTRPM2, activation by IDPR occurred as well, already at concentrations of 300 µM (Fig. 6c,d). However, the kinetics of the IDPR-induced currents were remarkably different to those in the presence of ADPR. In contrast to the almost immediate onset of currents when ADPR (150 µM) is infused into the cell through the patch pipette (see Figs 1c and 7a), a delay of at least 1 min (79.5 ± 19 s, n = 15, in comparison to 1.95 ± 0.36 s, n = 10, in the ADPR controls; p < 0.001) was consistently observed when IDPR (300 µM) was the stimulus (see also Fig. 7a). After onset, the current reached a peak rapidly as with ADPR. However, the following inactivation was considerably faster (see Figs 1c and 6c). The time after peak, over which the current was inactivated by 90% amounted to 10.9 ± 4 s, n = 26, as compared to 28 ± 14 s, n = 10 in the ADPR controls (p < 0.01). These kinetics are reminiscent of those of various NUDT9H-defective NvTRPM2 channel variants when stimulated with hydrogen peroxide (H_2_O_2_)^5,10^. The latency of current onset for H_2_O_2_-stimulated currents was 94 ± 9 s, n = 12 (see also Fig. 7a) and the time over which 90% of the current declined amounted to 9.2 ± 2.7 s, n = 11.

Reduced concentrations of IDPR (150 µM) failed to activate NvTRPM2 (Fig. 6d; n = 10). When we increased the concentration of IDPR to 600 µM, only marginal effects were observed on current amplitudes and kinetics compared with 300 µM (Figs 6d and 7a). However, at 1 mM, the delay of current onset was dramatically reduced to 6.1 ± 1.3 s (*n* = 7; p < 0.01; see also Fig. 7a).

### IDPR-dependent activation of NvTRPM2 by competitive inhibition of the NUDT9H-domain

The peculiar kinetics of IDPR-induced currents in NvTRPM2, which are mirrored by H_2_O_2_-dependent currents in NUDT9H-defective variants of NvTRPM2, give rise to the suspicion that they are related to a reduced ADPR-degrading capacity of the enzymatically active NUDT9H domain. As a definitive test for an indirect, NUDT9H-mediated action of IDPR, we repeated the experiments on the NvTRPM2-ΔNUD variant. Indeed, no activating effect was observed for any IDPR concentration up to 1 mM (Fig. 7b–d). Thus, IDPR does not activate NvTRPM2 through the second binding site. Moreover, it has no antagonistic effect on this site, because further experiments on NvTRPM2-ΔNUD revealed that IDPR (600 µM) did not impede the characteristic time course of ADPR-induced currents, neither during the co-application of ADPR (75 µM) via the patch-pipette (inset of Fig. 7c), nor after extracellular stimulation with H_2_O_2_ (Fig. 7d).

**Figure 8:**
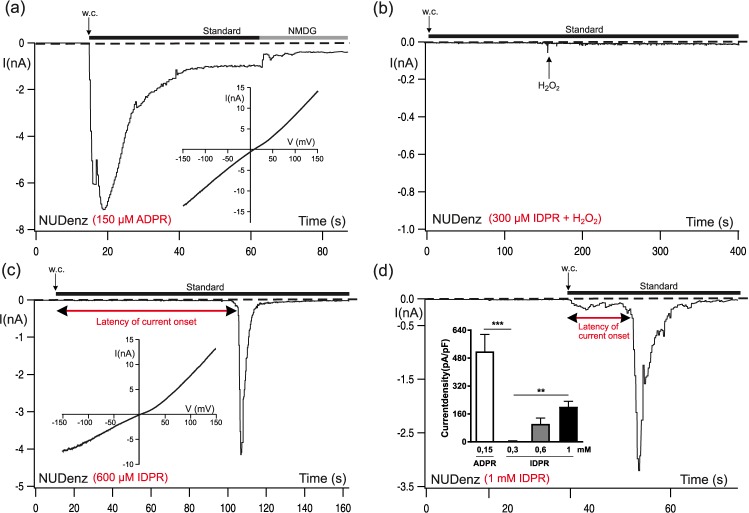
Effects of high concentrations of IDPR on the NvTRPM2-variant NUDenz. (**a**) In a control experiment, a HEK-cell expressing NvTRPM2-NUDenz was stimulated with 150 µM ADPR + 1 µM Ca^2+^. The corresponding current-voltage relation is given in the inset. (**b**) Absence of currents under stimulation with IDPR (300 µM). Even the subsequent extracellular application of H_2_O_2_ (10 mM, indicated by an arrow) failed to induce currents. (**c**) The increase of the intracellularly applied concentration of IDPR to 600 µM evoked characteristic currents with a pronounced latency in the onset (as indicated by the red double arrow) and rapid inactivation kinetics. The corresponding current-voltage relation is given in the inset. (**d**) A further increase of the applied concentration of IDPR concentration (1 mM) induced a considerable reduction of the latency of current onset (as indicated). The statistic of current densities in response to various concentrations of ADPR and IDPR (as indicated) obtained from whole-cell patch-clamp recordings of HEK-cells transfected with NvTRPM2-NUDenz is given in the inset. All data were presented as mean ± s.e.m. Differences are significant at **P < 0.01 and ***P < 0.001 evaluated with a one-way ANOVA and the Bonferroni correction, n = 5–8.

To provide further evidence that the enzymatic activity of NUDT9H is decisive for the indirect effect of IDPR on NvTRPM2, we used a channel chimera of NvTRPM2 in which the *C*-terminal NUDT9H domain was replaced by the corresponding sequence of the native human NUDT9 enzyme^5^. This manipulation is expected to alter strikingly the ADPRase activity, although the direction of the change cannot be predicted in advance. In any case, it should affect the dose-dependency of IDPR effects. As an initial control, we verified that NvTRPM2-NUDenz shows ADPR-dependent currents (Fig. 8a) almost indistinguishable from those elicited in the wild-type NvTRPM2 channel (Fig. 1c). In the presence of 300 µM IDPR in the patch pipette, no currents were elicited, not even after the additional stimulation with 10 mM extracellular H_2_O_2_ (Fig. 8b). Typical currents were induced by higher IDPR concentrations (n = 5 out of 8 with 600 µM and n = 6 out of 6 experiments with 1 mM). Again, these currents developed rapidly and showed fast inactivation (Fig. 8c,d). Additionally, increasing concentrations of IDPR shortened the time to the current onset profoundly from 62 ± 21 s at 600 µM IDPR (n = 4) to 15 ± 3 s at 1 mM IDPR (n = 6, p < 0.05) (see Fig. 8c,d). Thus, there was a shift in the dose-response-relationship towards significantly higher concentrations of IDPR in the NvTRPM2-NUDenz chimera when compared to wild-type NvTRPM2; higher concentrations of the activator were needed to reach the activation threshold and for comparably short delays in current onset. Moreover, the current density was reduced to roughly half (see Fig. 6d and inset of 8d). For further evidence of the indirect IDPR effect, we studied HEK-293 cells co-transfected with wild-type NvTRPM2 channel and hNUDT9-ADPRase (Supplementary-Fig. 1). Stimulation with 300 µM IDPR via the patch-pipette failed to induce significant currents in co-transfected HEK-cells (n = 7) whereas typical currents were elicited with 300 µM IDPR in controls, i.e. HEK-cells expressing wild-type NvTRPM2 alone (n = 6, p < 0.001). After doubling the IDPR concentration to 600 μM, typical currents consistently appeared also in HEK-cells co-expressing NvTRPM2 and hNUDT9 (n = 7) but were again significantly smaller than in controls (n = 7, p < 0.001).

## Discussion

In the present study, we have evaluated various ADPR derivatives with respect to their effects on the NUDT9H-independent ADPR binding site of the TRPM2 orthologue from the sea anemone *Nematostella vectensis*. In view of the recent cryo-electron microscopy study on DrTRPM2^5^ and the homology between zebra fish and sea anemone TRPM2, the term “*N*-terminal binding site” seems justified. This site mediates channel activation independently of the conventional ADPR binding site localized within the *C*-terminal ADPRase domain NUDT9H^10^ and interacts differently with its substrate.

We have determined distinct differences in the pharmacological profile of both sites. Three ADPR-analogues evaluated here evoked opposite effects on NvTRPM2 compared to hTRPM2. Two of them represent reported antagonists of hTRPM2 and in this study were also found to be agonists at NvTRPM2. The third one (IDPR), on the other hand, was the only one that was active on the human NUDT9 enzyme^33^ and on the full-length channels of hTRPM2 and NvTRPM2 alike, but not on the corresponding channel variants without NUDT9H domain. Thus, IDPR does not affect the *N*-terminal interaction site but acts exclusively on the C-terminal one. This is remarkable, for two reasons. First, IDPR is structurally much more similar to ADPR than the analogues 8-(thiophen-3-yl)-ADPR and 8-(3-acetyl-phenyl)-ADPR, but nevertheless discriminates between the two binding sites. Second, the pharmacological and functional differences between these sites allow a straightforward explanation of the indirect mechanisms by which IDPR induces gating of NvTRPM2; these findings furthermore elucidate the opposite roles that the NUDT9H domain plays in the TRPM2 channel orthologues of man and sea anemone, respectively.

The primary structure of the NUDT9H domain in NvTRPM2 is much more similar to the native NUDT9 enzyme than the NUDT9H domains of hTRPM2 or DrTRPM2. In particular, the signature of the catalytic center of NUDT9 is only conserved in NvTRPM2^5^. We have previously presented and outlined experimental evidence for a regulatory function of NUDT9H in NvTRPM2; specifically, the preserved ADPRase activity of the enzyme domain limits the availability of ADPR at the *N*-terminal binding site and thereby controls channel gating^10,11^ For our further reasoning, it is important to note that at higher concentrations (300 µM), IDPR is hydrolyzed by the human NUDT9H-ADPRase with a similar conversion rate to ADPR^33^. Therefore, the NUDT9H domain in NvTRPM2 should hydrolyze ADPR at a smaller rate when IDPR is present at higher concentrations. As a result, endogenous ADPR should accumulate over time until it reaches the threshold for activation of NvTRPM2 via the *N*-terminal ADPR binding site. The experimental results are completely in line with this view. In particular, there was always a marked delay before the abrupt onset of IDPR-dependent channel activation. This delay, in turn, was modified by IDPR in a concentration-dependent manner. Moreover, the dose-response-relationship of IDPR was shifted towards considerably higher concentrations in a variant of NvTRPM2 with altered ADPRase activity of the NUDT9H domain. The co-expression of the separate NUDT9-ADPRase led in principle to the same results. However, due to the uncertain stoichiometry of the two co-expressed proteins, quantitative statements are of limited reliability.

The indirect mechanism of NvTRPM2 activation outlined is the simplest interpretation of the data. More complex explanations could be imagined; especially, interactions of ADPR binding sites could be envisaged which result in mutual regulation and modification by particular ligands. A similar situation exists for the activation mechanisms of H_2_O_2_^5,10^, which leads to the intracellular release of ADPR^8^. In NvTRPM2, H_2_O_2_ becomes a channel activator only if the ADPR-degrading function of the endogenous NUDT9H domain of NvTRPM2 is experimentally disrupted^5,10^. The proposed indirect way of stimulation, which might be very close to the natural conditions, is well compatible with the characteristic “all-or-nothing” activation mode of NvTRPM2, but again the view may have to be extended when more information about the interaction of ADPR binding sites becomes available.

Although primarily focused on NvTRPM2, the present study also demonstrates for hTRPM2 that high concentrations of IDPR were able to activate hTRPM2 in the same way as ADPR. The fact that the required concentrations of IDPR were dramatically higher than those of ADPR indicates an inefficient binding and reduced occupancy of IDPR at the NUDT9H domain. Further detailed electrophysiological analyses are necessary to get more information how this affects channel gating. Since IDPR fails to stimulate currents in hTRPM2 channel mutants without a NUDT9H domain, evidence exists that not only ADPR but also IDPR binds to this domain as a prerequisite to gating. The findings especially on ADPR are in line with the cryo-EM study on hTRPM2^17^, whereas contrasting results in DrTRPM2 demonstrating ADPR binding exclusively in *N*-terminus^15^ represent species differences in all likelihood. Apparently, the roles of the N-terminus and the C-terminus are opposite in hTRPM2 on the one side and in DrTRPM2 and NvTRPM2 on the other.

The characterization of the pharmacological profile of the second ADPR binding site in NvTRPM2 is certainly of value on its own. More importantly, it may be the basis that enables further characterization of the *N*-terminal binding pocket (Huang *et al*.,)^15^. Possible experimental strategies include consecutive mutations of candidate amino acid residues in the three TRPM2 orthologues, combined with the determination of the resulting pharmacological profile, i.e. the responses to various ADPR analogues, notably IDPR.

Another important aspect of the study relates to the biological role of NUDT9H in NvTRPM2. So far, its function has been deduced from mutations and deletions; now, pharmacological evidence is added in wild-type channels by the experiments with IDPR. Although a physiological function of IDPR has not yet been described in the literature, inosine has been identified as a release product of dead and dying cells^34^. It is conceivable that inosine or inosine-containing compounds like IDPR might play a so-far-undiscovered crucial role in organisms like *Nematostella vectensis*. If IDPR cannot achieve such a role because it is not present in sufficient concentrations, there may be other factors that modify the associated ADPR-degrading function of NvTRPM2. The cellular control of this apparent auto-regulatory function sets the availability of ADPR for the second binding site and thereby determines the channel activity of NvTRPM2 that may be part of several signaling cascades employing its depolarizing and Ca^2 + ^-elevating properties. Even for hTRPM2, it has been proposed that an NUDT9H-coupled ADPRase exerts a control of ADPR availability in the hippocampus^35^.

In conclusion, the determination of the pharmacological profile of the NUDT9H-independent ADPR binding site of NvTRPM2 may provide indispensable tools for a universally applicable molecular model of the novel ADPR effector site within the *N*-terminus of TRPM2 channels. At the same time, it enables us to understand and differentiate the distinct roles that the two binding sites of NvTRPM2 play and how they cooperate in the regulation and control of channel activity.

## Methods

### Molecular cloning

The cDNAs of human TRPM2 as well as of the TRPM2 channel variants of *Nematostella vectensi*: full-length (wild-type), NUDT9H-truncated (NvTRPM2-∆NUD) and NUDT9-substituted (NvTRPM2-NUDenz) were generated and subcloned as describe elsewhere^5,10^. The co-expression of wild-type NvTRPM2 with human NUDT9 ADPRase was performed as previously described^10^.

### ADPR-analogues

Synthetic ADPR analogues were prepared as follows; inosine 5′-diphosphate ribose (IDPR) and 2-fluoro-adenosine 5′-diphosphate ribose (2F-ADPR) were synthesized by NADase hydrolysis of their respective linear precursors nicotinamide hypoxanthine 5′-diphosphate ribose (NHD^+^) and nicotinamide 2-fluoro-adenosine 5′-diphosphate ribose (2F-NAD^+^) respectively^25^; 8-bromo adenosine 5′-diphosphate ribose (8-bromo-ADPR) was prepared by treatment of commercially available NAD^+^ with NADase followed by bromination; subsequent Suzuki reactions gave 8-(thiophen-3-yl)-adenosine 5′-diphosphate ribose (8-TP-ADPR), and 8-(3-acetyl-phenyl)-adenosine 5′-diphosphate ribose (8-(3-AP)-ADPR)^27^; β-cyclopentyl-adenosine 5′-diphosphate ribose (β-cyclopentyl-ADPR) was synthesized as described^27^; β-methyl-adenosine 5′-diphosphate ribose (β-methyl-ADPR) was synthesized as described^13^ and adenosine 5′-diphosphate–α-D-glucoside (ADP-glucose) was obtained from Sigma-Aldrich. The purity of compounds evaluated was >95% as determined by analytical HPLC analysis, generally carried out on a Waters 2695 Alliance module equipped with a Waters 2996 Photodiode Array Detector (210–350 nm). The chromatographic system consisted of a Hichrom Guard Column for HPLC and a Phenomenex Synergi 4 u MAX-RP 80 A column (150 × 4.60 mm), eluted at 1 mL/min with the following ion-pair buffer: 0.17% (m/v) cetrimide and 45% (v/v) phosphate buffer (pH 6.4) in MeOH.

### Cell culture and transfection

Human embryonic kidney (HEK-293) cells were obtained from the German Collection of Microorganisms and Cell Cultures (Braunschweig, Germany) and cultured in DMEM media (Biochrome, Berlin, Germany) supplemented with 4 mM L-glutamine, 10% (v/v) foetal calf serum (Biochrome) and 2 mM sodium pyruvate. Transient transfections of HEK-293 cells with the cDNAs of wild-type hTRPM2 or NvTRPM2 variants were performed using the FuGene 6 transfection reagent (Roche, Mannheim, Germany) according to the manufacturer’s instructions. In some control experiments the human NUDT9 ADPRase was co-expressed as previously described^10^. The transfected cells were maintained for 24 h in an incubator at 37 °C and 5% CO_2_. Subsequently, the cells were seeded on poly-lysine-coated glass coverslips at a suitable dilution and further incubated for 3–4 h. Then patch-clamp experiments were carried out in cells visibly positive for EGFP (and additionally DsRed in co-expression experiments). At least three independent transfections were used for each experimental group.

### Electrophysiology

Whole-cell recordings were performed using an EPC 9 amplifier equipped with a personal computer with Pulse 8.5 and X Chart software (HEKA, Lamprecht, Germany). The standard bath solution contained (in mM) 140 NaCl, 1.2 MgCl_2_, 1.2 CaCl_2_, 5 KCl, 10 HEPES, pH 7.4 (NaOH). For Na^+^ free solutions, Na^+^ was replaced by 150 mM N-methyl-D-glucamine (NMDG) and the titration was performed with HCl. The pipette solution contained (in mM) 145 CsCl, 8 NaCl, 2 MgCl_2_, 10 HEPES, pH 7.2 (CsOH) and the Ca^2+^ concentration was adjusted to 1 µM (0.886 mM Ca^2+^, 1 mM Cs-EGTA).

The Ca^2+^ concentration of the solutions was calculated using the *MAXC*-program: (http://www.stanford.edu/~cpatton/maxc.html). For the stimulation of TRPM2, adenosine 5′-diphosphate ribose (ADPR, Sigma-Aldrich, Munich, Germany) as well as the ADPR analogues were prepared as 100 mM stock solutions in distilled water and aliquots stored at −20 °C. Each of these substances was diluted to the desired concentration in the intracellular (pipette) solution on the day of the experiment. Unless otherwise stated, the experiments were performed at room temperature (21 °C) and the current-voltage relations were obtained during voltage ramps from −150 to + 150 mV and back to −150 mV applied over 200 ms. The holding potential was −60 mV. For the analysis the maximal current amplitudes (pA) in a cell were divided by the cell capacitance (pF), as measure of the cell surface, resulting in the current density (pA/pF).

### Data analysis and statistics

Graphs were generated using CorelDraw 12, IgorPro 5.01 and GraphPad Prism 6. Data are expressed as the mean ± s.e.m. Unless stated otherwise, the comparison of two groups was performed using an unpaired Student’s t-test. One-way ANOVA with Bonferroni correction was applied when multiple comparisons were performed with the same control data. Differences were considered significant at *P < 0.05, **P < 0.01 and ***P < 0.001. n.s., not significant.

## Supplementary information

Supplementary Info
