# Supplementary material for: Different substrate specificities of the two ADPR binding sites in TRPM2 channels of Nematostella vectensis and the role of IDPR
Source: Sci Rep. 2019 Mar 21;9:4985. doi: 10.1038/s41598-019-41531-4 (PMC6428886; doi:10.1038/s41598-019-41531-4)
Supplement: Supplementary file 1 — Supplementary Info [file 41598_2019_41531_MOESM1_ESM.pdf]

SUPPLEMENTARY INFORMATION

ARTICLE TITLE: Different substrate specificities of the two ADPR binding sites in TRPM2 channels of *Nematostella vectensis* and the role of IDPR

AUTHORS: Frank J. P. Kühn[1*], Joanna M. Watt[2,3], Barry V. L. Potter[2] & Andreas Lückhoff[1]

[*]Corresponding author: PD Dr. Frank Kühn, Institute of Physiology, RWTH Aachen. Email: fkuehn@ukaachen.de. Telephone +49 (0) 241 8088803

AUTHOR AFFILIATION: [1]Institute of Physiology, Medical Faculty, RWTH Aachen, D52057 Aachen, Germany. [2]Medicinal Chemistry and Drug Discovery, Department of Pharmacology, University of Oxford, Mansfield Road, Oxford, OX1 3QT, UK. [3]Wolfson Laboratory of Medicinal Chemistry, Department of Pharmacy and Pharmacology, University of Bath, Claverton Down, Bath, BA2 7AY, UK.

SUPPLEMENTARY INFORMATION LEGEND

Figure S1. Coexpression of human NUDT9 ADPRase decreases the sensitivity of wild-type NvTRPM2 to IDPR

Whole-cell patch-clamp experiments of HEK-293 cells, either co-expressing wild-type NvTRPM2 and human NUDT9 enzyme **(a** and **b)** or wild-type NvTRPM2 alone **(c)**. Co-expression was performed as described previously[10] using human NUDT9 enzyme (DsRed as expression marker) and wild-type NvTRPM2 (EGFP as expression marker). Stimulation was performed with 1 µM Ca^{2+} and different concentrations of IDPR in the patch-pipette (as indicated). The corresponding I/V relations are given as insets. **(d)** Summary of the effects of IDPR on co-expression of human NUDT9 enzyme. All data were presented as mean ± s.e.m..

Differences are significant at *** ($P < 0.001$) evaluated with the unpaired Student's t-test, $n \geq 6$.

Figure-S1

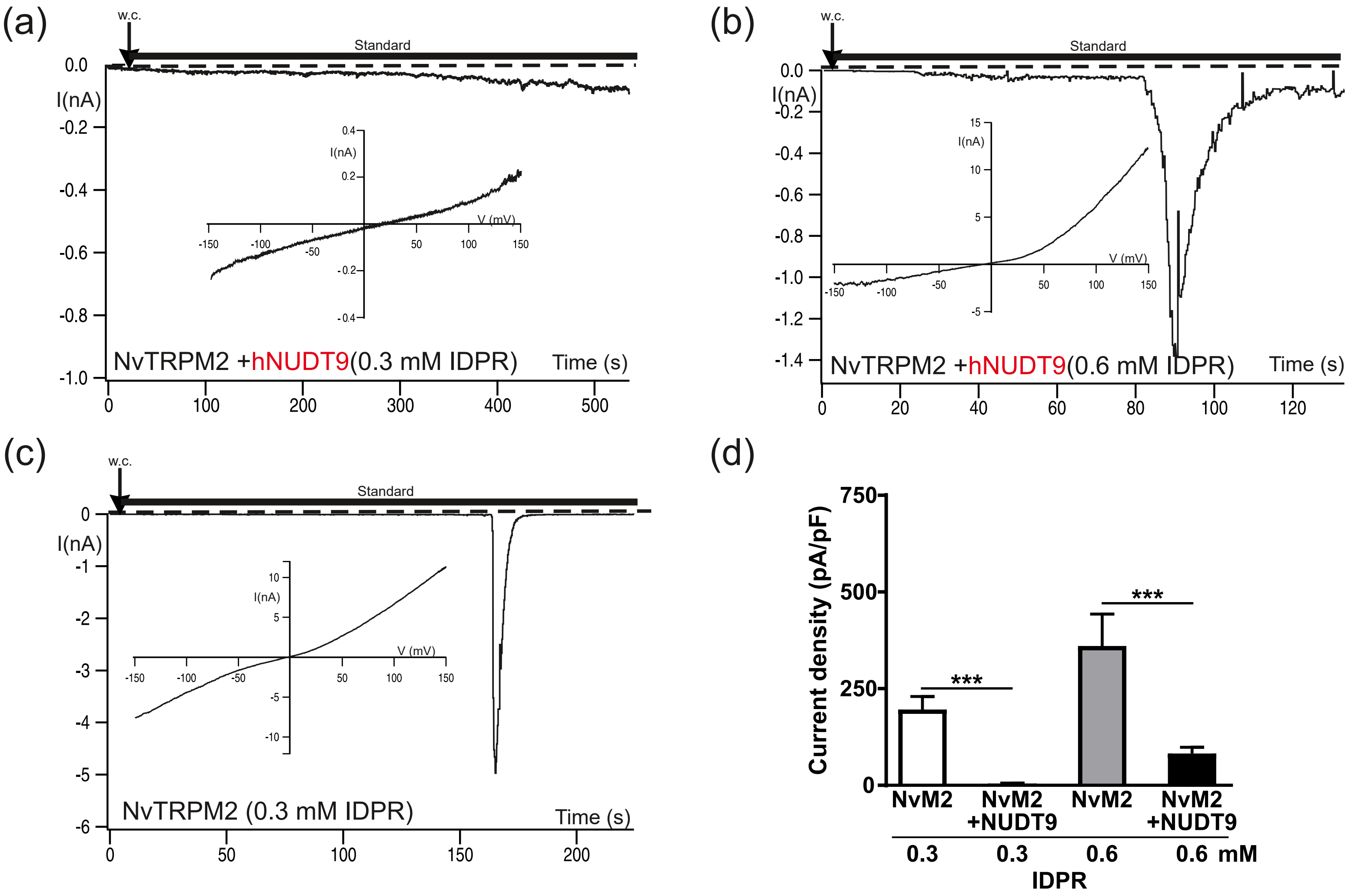

(a)
w.c.
Standard
I(nA)
NvTRPM2 +hNUDT9(0.3 mM IDPR)
Time (s)
V (mV)
(b)
w.c.
Standard
I(nA)
NvTRPM2 +hNUDT9(0.6 mM IDPR)
Time (s)
V (mV)
(c)
w.c.
Standard
I(nA)
NvTRPM2 (0.3 mM IDPR)
Time (s)
V (mV)
(d)
Current density (pA/pF)
***
***
NvM2
NvM2 +NUDT9
NvM2
NvM2 +NUDT9
0.3
0.3
0.6
0.6 mM
IDPR
